# The prognostic value of the preoperative albumin to alkaline phosphatase ratio in patients with non‐small cell lung cancer after surgery

**DOI:** 10.1111/1759-7714.13107

**Published:** 2019-06-03

**Authors:** Lianmin Zhang, Hua Zhang, Dongsheng Yue, Wei Wei, Yulong Chen, Xiaoliang Zhao, Jianquan Zhu, Bin Zhang, Zhenfa Zhang, Changli Wang

**Affiliations:** ^1^ Department of Lung Cancer, Tianjin Lung Cancer Center, Tianjin Medical University Cancer Institute and Hospital, National Clinical Research Center for Cancer, Key Laboratory of Cancer Prevention and Therapy Tianjin's Clinical Research Center for Cancer Tianjin China

**Keywords:** Albumin to alkaline phosphatase ratio, non‐small cell lung cancer, prognosis

## Abstract

**Background:**

To assess the potential prognostic value of the albumin to alkaline phosphatase ratio (AAPR) in patients with non‐small cell lung cancer (NSCLC) after surgery.

**Methods:**

The log‐rank and Kaplan‐Meier analyses were performed to detect differences in survival levels between different groups. A model of Cox proportional hazards was used to perform univariate and multivariate survival analyses. Comparisons of receiver operating characteristic (ROC) curves and the likelihood ratio test (LRT) were also utilized to compare the prognostic abilities of different systems for overall survival (OS) prediction.

**Results:**

The optimal cut‐off value of the preoperative AAPR was 0.64. A decreased AAPR was associated with several clinicopathological and clinicolaboratory variables related to cancer progression. The preoperative AAPR of patients was positively correlated with the poor prognosis of NSCLC. In multivariate analyses, the preoperative AAPR was identified as an independent prognostic factor for disease‐free survival (DFS; *P* = 0.001) and overall survival (OS; *P* = 0.003). The LRT showed that the AAPR tumor‐node‐metastasis (TNM) system presented a significantly larger χ^2^ value (112.4 vs. 89.2, respectively, *P* < 0.01) and a relatively smaller Akaike information criterion (AIC) value (2955 vs. 2977, respectively, *P* < 0.01) than the TNM staging system.

**Conclusion:**

Preoperative AAPR was a potentially valuable prognostic factor in NSCLC patients who underwent surgery. Our results further showed that the AAPR‐TNM system was superior to the current TNM staging system.

## Introduction

Lung cancer is one of the most common malignancies and has a significantly high mortality rate. In 2018, approximately 2.1 million people were diagnosed with different stages of lung cancer, and nearly 1.7 million of these patients died.^1^ Despite the advances in multimodal treatment strategies in recent decades, the prognosis of lung cancer remains poor, with 5‐year survival rates of less than 15%. Additionally, although the tumor‐node‐metastasis (TNM) staging system is a critical and widely used tool for prognostic assessment,^2^ it fails to accurately predict the prognosis of some lung cancer patients. It is therefore of great importance to define novel and effective prognostic markers and therapeutic targets.

As a major component isolated from human serum, albumin (ALB) not only reflects the nutritional status of the body but also plays a critical role in the inflammatory response.^3^Previous studies demonstrated that the ALB level in serum could independently predict survival levels in several types of malignancies, such as colorectal cancer, breast cancer, ovarian cancer, gastric cancer and lung cancer.^4^, ^5^, ^6^ The levels of the hydrolase enzyme alkaline phosphatase (ALP) increase in cancer patients in association with bone metastasis; hence, ALP was used to screen patients for bone metastasis. The ALB to ALP ratio (AAPR) is the ratio of the serum ALB level to the ALP level. In 2015, Anthony *et al*. first reported the prognostic value of the AAPR and revealed that the AAPR was a significant prognostic predictor in hepatocellular carcinoma (HCC).^7^ Similar studies have been subsequently reported in other types of cancers,^8^, ^9^ including small cell lung cancer (SCLC).^10^ However, the prognostic value of the AAPR has not yet been well studied in non‐small cell lung cancer (NSCLC) patients. Here, we conducted a retrospective study to evaluate the clinical significance of the preoperative AAPR in the prognosis of NSCLC patients who underwent surgery.

## Methods

### Patients

A total of 567 NSCLC patients who underwent complete pulmonary resection (lobectomy or pneumonectomy) and systematic node dissection of the hilar and mediastinal lymph nodes at Tianjin Medical University Cancer Institute and Hospital between January 2006 and December 2010 were enrolled in this retrospective study. A total of 71 NSCLC patients were excluded from the study for the following reasons: received chemotherapy or radiotherapy before surgery treatment (*n* = 30), other concomitant malignancies (*n* = 8), positive surgical margins (*n* = 3), liver disease, autoimmune disease or bone disease that could affect ALB and ALP levels (*n* = 19), and missing data (*n* = 11). Finally, exhibit of the 567 patients satisfied our inclusion criteria and were enrolled in our present study. Preoperative evaluation included physical examination, blood laboratory tests, flexible bronchoscopy, chest and upper abdominal computed tomography (CT), brain magnetic resonance imaging or CT, and a radionuclide bone scan. All patients were restaged according to the eighth edition of the TNM classification.^2^ The histological subtypes were determined according to the 2015 WHO guidelines.^11^ In addition, this study was approved by the ethics committee of Tianjin Medical University Cancer Institute and Hospital. Informed consent was not required because of the retrospective nature of this study.

### Data collection and records

Baseline clinical pathological parameters were retrieved from the hospital database and reviewed. The following clinical data were classified for patients: age, sex, tumor location, smoking status, resection type, histological subtype, etc. Relevant laboratory data, including the platelet count (PLT), white blood cell count (WBC), levels of hemoglobin (Hb), D‐dimer, fibrinogen, ALP, and ALB, were also collected and analyzed 1 week before surgery. All patients were followed every threemonths for the first year and every sixmonths for the next 3 years.

### Definition of the cut‐off value of the AAPR

**Figure 1:**
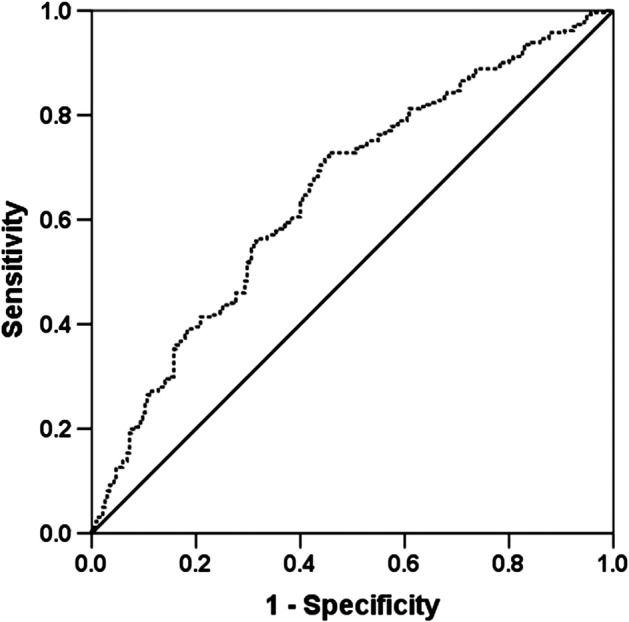
ROC analysis of the preoperative AAPR for 496 NSCLC patients. AUC = 0.652, 95% CI = 0.604–0.701.

The preoperative AAPR was defined as follows: AAPR = ALB level in serum/ALP level. The preoperative AAPR threshold value was evaluated by receiver operating characteristic (ROC) curve analysis and the Youden index (Youden index = sensitivity + specificity‐1). For the 496 NSCLC patients, an AAPR value of 0.64 corresponded to the maximum Youden index value. Thus, the recommended threshold value for the preoperative AAPR was 0.64. A total of 199 patients had an AAPR greater than or equal to 0.64, whereas 297 patients had an AAPR value less than 0.64, which was defined as low AAPR. The area under the curve (AUC) for the preoperative AAPR was 0.652 (95% confidence interval [CI]: 0.604–0.701) (Fig 1).

### Statistical analysis

All statistical analyses in this study were performed using SPSS 22.0 software (Inc., Chicago, IL). A ROC curve was used to evaluate the use of the value when the Youden index reached the maximum value as the optimal threshold value of the AAPR.^12^ According to the threshold value of the AAPR, all NSCLC patients were classified into two groups (low‐ and high‐AAPR groups). The potential correlations between the AAPR level and categorical variables or continuous variables were analyzed by chi‐square test or Mann‐Whitney U test, respectively. Kaplan‐Meier and log‐rank analyses were also performed to analyze the prognosis based on overall survival (OS) and disease‐free survival (DFS). The ROC curves and the likelihood ratio test (LRT) were calculated to compare the prognostic abilities for OS prediction between different systems. A *P‐*value less than 0.05 was regarded as statistically significant.

## Results

### Analysis of clinicopathological characteristics

The clinicopathological features of NSCLC patients were analyzed and are shown in Table 1. In brief, we collected 496 patients, most of whom were male (male/female: 334/162). The median age of the cohort at presentation was 60 yearsold (range: 34–81 years old). There were 297 patients whose AAPR value was less than 0.64 and 199 patients with an AAPR value ≥0.64. Based on the eighth edition of the TNM classification system, the TNM distribution of patients was 181 patients in stage I, 122 in stage II, and 193 in stage III. The median follow‐up duration was 47.0 months (range from 2.0 months to 96.0 months). Furthermore, 235 patients (47.4%) survived, but only 161 patients (32.4%) did not experience tumor relapse. The 5‐year total survival rate was 50.4% for all recorded NSCLC patients.

**Table 1:** The relationship between the preoperative AAPR and clinicopathological variables

Variables	AAPR<0.64 (*n* = 297)	AAPR≥0.64 (*n* = 199)	*P*‐value
Age			
≤60	150 (57.0%)	113 (43.0%)	
>60	147 (63.1%)	86 (36.9%)	0.170
Sex			
Female	96 (59.3%)	66 (40.7%)	
Male	201 (60.2%)	133 (39.8%)	0.845
Smoking status			
Yes	208 (62.5%)	125 (37.5%)	
No	89 (54.6%)	74 (45.4%)	0.093
Resection type			
Pneumonectomy	29 (52.7%)	26 (47.3%)	
Lobectomy	268 (60.8%)	173 (39.2%)	0.251
Tumor location			
Left	119 (59.8%)	80 (40.2%)	
Right	178 (59.9%)	119 (40.1%)	0.976
Lesion			
Peripheral	208 (57.9%)	151 (42.1%)	
Central	89 (65.0%)	48 (35.0%)	0.154
Histological subtype			
SqCC	158 (65.6%)	83 (34.4%)	
Adenocarcinoma	103 (51.5%)	97 (48.5%)	
Others	36 (65.4%)	19 (34.6%)	0.007
T stage			
T1	88 (48.4%)	94 (51.6%)	
T2	120 (62.2%)	73 (37.8%)	
T3	50 (67.6%)	24 (32.4%)	
T4	39 (83.0%)	8 (17.0%)	<0.001
Lymph node metastasis			
Yes	139 (66.5%)	70 (33.5%)	
No	158 (55.0%)	129 (45.0%)	0.010
TNM stage			
I	88 (48.6%)	93 (51.4%)	
II	77 (63.1%)	45 (36.9%)	
III	132 (68.4%)	61 (31.6%)	<0.001

AAPR, albumin to alkaline phosphatase ratio; SqCC, squamous cell carcinoma.

### Correlation analysis between preoperative AAPR and clinical pathological or clinical laboratory variables

The relationships between the preoperative AAPR and clinical pathological variables were then analysed, and the results are shown in Table 1. According to the analysis, significant differences between the low and high preoperative AAPR groups were identified for histological subtype (*P* = 0.007), T stage (*P* < 0.001), lymph node metastasis (LNM) status (*P* = 0.010), and pathological TNM stage (*P* < 0.001). Table 2 further exhibited the correlations between the preoperative AAPR and clinical laboratory variables. According to the results, the preoperative AAPR was significantly associated with tumor diameter (*P* < 0.001), PLT (*P* = 0.024), WBC count (*P* = 0.001), LDH level (*P* < 0.001), D‐dimer level (*P* = 0.001), fibrinogen level (*P* < 0.001), ALB concentration (*P* < 0.001), and ALP level (*P* < 0.001).

**Table 2:** The relationship between preoperative AAPR and clinicolaboratory variables

Variables	AAPR<0.64 (*n* = 297)	AAPR≥0.64 (*n* = 199)	*P‐* value
Age (year)	60.9 ± 8.6	59.6 ± 9.5	0.139
Maximum tumor diameter (cm)	4.7 ± 2.2	3.9 ± 2.0	<0.001
Platelet count (× 10^4^ mm^−3^)	25.6 ± 7.6	24.1 ± 6.6	0.024
WBC count (× 10^3^ mm^−3^)	7.0 ± 1.7	6.6 ± 1.7	0.001
Hb (g/L)	138.9 ± 14.3	139.4 ± 15.5	0.302
LDH (U/L)	191.1 ± 60.6	169.1 ± 33.4	<0.001
D‐dimer (mg/L)	0.3 ± 0.2	0.2 ± 0.2	0.001
Fibrinogen (g/L)	3.9 ± 1.0	3.3 ± 0.8	<0.001
ALB (g/dl)	4.2 ± 0.4	4.4 ± 0.4	<0.001
ALP (U/L)	90.3 ± 25.7	56.4 ± 9.9	<0.001

AAPR, albumin to alkaline phosphatase ratio; ALB, albumin; ALP, alkaline phosphatase; Hb, hemoglobin; LDH, lactate dehydrogenase; WBC, white blood cell.

### Analysis of the association between preoperative AAPR and patient survival

To further assess the potential prognostic value, survival analyses in relation to the preoperative AAPR and patient prognosis were subsequently conducted. In the univariate analyses of DFS and OS, the lesion type (*P* = 0.016 and *P* = 0.020, respectively), resection type (*P* = 0.018 and *P* = 0.024, respectively), PLT (both are *P* = 0.006), Hb level (*P* = 0.006 vs. *P* = 0.008), fibrinogen level (both are *P* < 0.001), LDH level (*P* < 0.001 and *P* = 0.001), D‐dimer level (*P* = 0.003 vs. *P* = 0.031), ALB level (*P* = 0.001 vs. *P* = 0.002), ALP level (both are *P* < 0.001), AAPR (both are *P* < 0.001), and TNM stage (both are *P* < 0.001) were thought to be obvious factors (Table 3).

**Table 3:** Univariate analysis of DFS and OS for all NSCLC patients

	DFS			OS		
	*P*‐value	HR	95% CI	*P‐*value	HR	95% CI
Age (≤60, >60)	0.591	1.069	0.838 – 1.363	0.526	1.082	0.849 – 1.379
Sex (female, male)	0.303	1.143	0.886 – 1.476	0.530	1.085	0.841 – 1.401
Smoking status (yes, no)	0.966	1.006	0.777 – 1.302	0.527	0.920	0.710 – 1.192
Resection type (pneumonectomy, lobectomy)	0.018	1.524	1.076 – 2.158	0.024	1.493	1.054 – 2.114
Tumor location (left, right)	0.210	0.855	0.669 – 1.092	0.208	0.854	0.669 – 1.092
Lesion (peripheral, central)	0.016	1.379	1.063 – 1.790	0.020	1.364	1.051 – 1.770
Histological subtype (squamous, adenocarcinoma, others)	0.653	0.959	0.799 – 1.151	0.530	0.944	0.787 – 1.131
TNM stage (I, II, III)	<0.001	1.958	1.679 – 2.284	<0.001	1.930	1.655 – 2.252
Platelet count (× 10^4^ mm^−3^)	0.006	1.458	1.114 – 1.908	0.006	1.458	1.115 – 1.908
WBC count (× 10^3^ mm^−3^)	0.057	1.287	0.993 – 1.669	0.081	1.260	0.972 – 1.634
Hb (g/L)	0.006	0.691	0.529 – 0.901	0.008	0.697	0.534 – 0.910
LDH (U/L)	<0.001	2.134	1.458 – 3.123	0.001	1.949	1.331 – 2.852
Fibrinogen (g/L)	<0.001	1.551	1.216 – 1.979	<0.001	1.594	1.249 – 2.035
D‐dimer (mg/L)	0.003	1.448	1.134 – 1.849	0.031	1.310	1.024 – 1.675
ALB (g/dl)	0.001	0.628	0.476 – 0.828	0.002	0.649	0.492 – 0.857
ALP (U/L)	<0.001	1.694	1.323 – 2.168	<0.001	1.683	1.315 – 2.154
AAPR (≥0.64, <0.64)	<0.001	0.459	0.350 – 0.603	<0.001	0.466	0.355 – 0.611
TNM stage (I, II, III)	<0.001	1.958	1.679 – 2.284	<0.001	1.930	1.655 – 2.252

AAPR, albumin‐to‐alkaline phosphatase ratio; ALB, albumin; ALP, alkaline phosphatase; CI, confidence interval; DFS, disease‐free survival; Hb, hemoglobin; HR, hazard ratio; LDH, lactate dehydrogenase; OS, overall survival; WBC, white blood cell.

**Figure 2:**
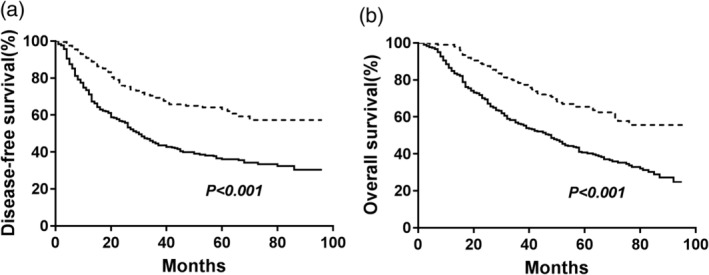
Kaplan‐Meier analysis of DFS and OS stratified by preoperative AAPR value in patients with NSCLC. (**a**) Effect of AAPR on DFS; (**b**) Effect of AAPR on OS. *P‐*values were calculated by the log‐rank test. (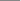) <0.64 and (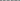) ≥0.64.

**Figure 3:**
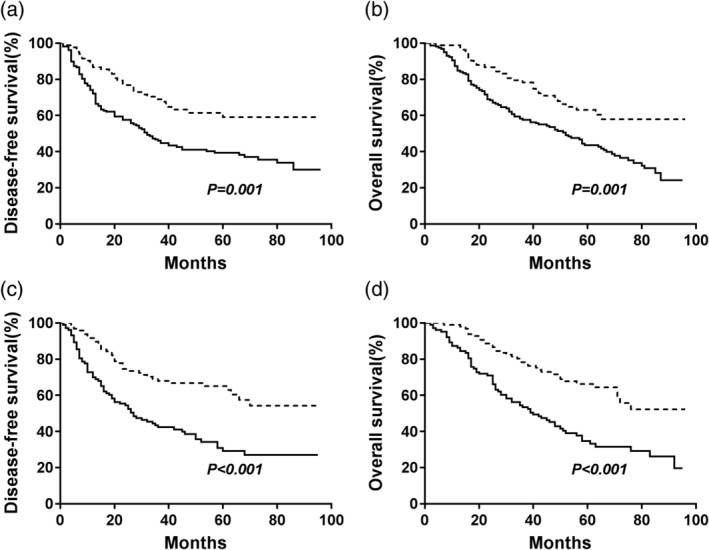
Kaplan‐Meier analysis of DFS and OS stratified by preoperative AAPR value in different histological subtypes. (**a**) Effect of AAPR on DFS in lung squamous cell carcinoma (SqCC). (**b**) Effect of AAPR on OS in lung squamous cell carcinoma (SqCC). (**c**) effect of AAPR on DFS in lung adenocarcinoma. (**d**) effect of AAPR on OS in lung adenocarcinoma. *P‐*values were calculated by the log‐rank test. (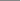) <0.64 and (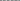) ≥0.64.

**Figure 4:**
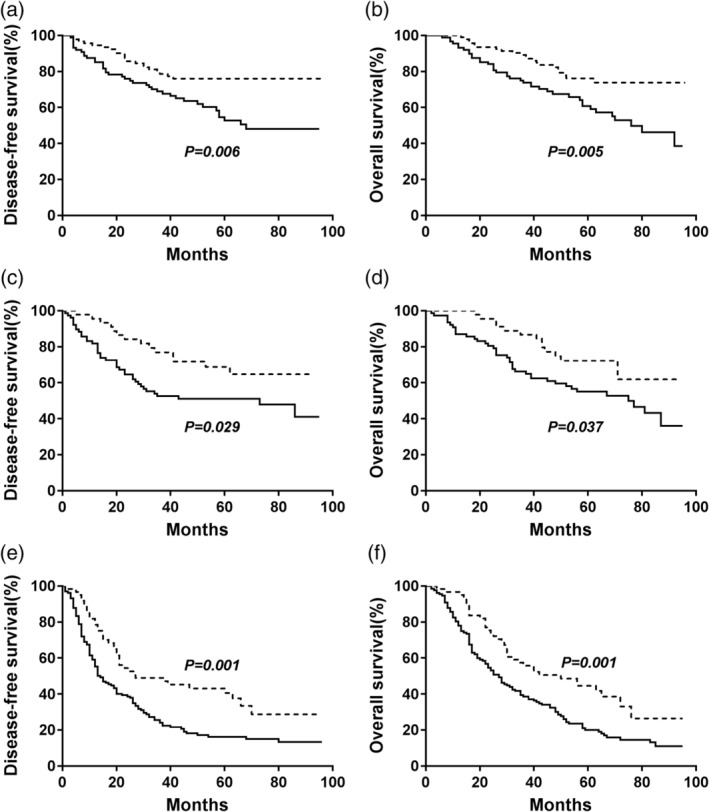
Kaplan‐Meier curves of DFS and OS stratified by preoperative AAPR value in different TNM stages. (**a**) Effect of AAPR on DFS in stage I. (**b**) Effect of AAPR on OS in stage I. (**c**) Effect of AAPR on DFS in stage II at the beach. (**d**) Effect of AAPR on OS in stage II. (e) Effect of AAPR on DFS in stage III. (f) Effect of AAPR on OS in stage III. *P‐*values were calculated by the log‐rank test. (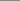) <0.64 and (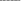) ≥0.64.

The median survival time and the 5‐year DFS, which reflects patient prognosis in the high‐AAPR (equal to or greater than 0.64) group were obviously higher than those in the low‐AAPR (less than 0.64) group (median survival: 43.0 months vs. 29.0 months; DFS rate: 63.1% vs. 36.1%, respectively, *P* < 0.001, Fig 2a). The median survival time was 52.0 months for the group with AAPR greater than or equal to 0.64 and 41.0 months for the group with AAPR less than 0.64, and the 5‐year OS rates were 65.5% and 40.2%, respectively (*P* < 0.001, Fig 2b). Subgroup analyses showed that patients with an AAPR greater than or equal to 0.64 had better DFS and OS regardless of their histological subtype (Fig 3). When the analysis was stratified by pathological TNM staging, our results demonstrated that DFS and OS were poorer in the low‐AAPR group than in the high‐AAPR group in the pathological stage I, II, and III subgroups (stage I: *P* = 0.006 for DFS, *P* = 0.005 for OS, Fig 4a,b; stage II: *P* = 0.029 for DFS, *P* = 0.037 for OS, Fig 4c,d; and stage III: *P* = 0.001 for DFS, *P* = 0.001 for OS, Fig 4e, f).

To further assess independent prognostic indicators, 11 clinicopathological characteristics that were significant in the univariate analysis were enrolled in the multivariate analysis (Table 4). Notably, multivariate analysis further demonstrated that the preoperative AAPR was an independent prognostic indicator, including DFS (HR: 0.510, 95% CI: 0.338–0.770, *P* = 0.001) and OS (HR: 0.536, 95% CI: 0.364–0.818, *P* = 0.003).

**Table 4:** Multivariate analysis of DFS and OS for all NSCLC patients

	DFS			OS		
	*P*‐value	HR	95% CI	*P*‐value	HR	95% CI
Resection type (pneumonectomy, lobectomy)	0.716	1.079	0.716 – 1.625	0.776	1.061	0.705 – 1.596
Lesion (peripheral, central)	0.222	1.203	0.894 – 1.620	0.429	1.128	0.837 – 1.518
Platelet count (× 10^4^ mm^−3^)	0.355	1.147	0.858 – 1.534	0.267	1.178	0.883 – 1.572
Hb (g/L)	0.100	0.783	0.585 – 1.048	0.087	0.776	0.580 – 1.037
LDH (U/L)	0.044	1.512	1.011 – 2.261	0.229	1.284	0.855 – 1.927
D‐dimer (mg/L)	0.253	1.162	0.898 – 1.504	0.532	1.086	0.839 – 1.404
Fibrinogen (g/L)	0.793	0.965	0.740 – 1.259	0.879	1.021	0.782 – 1.333
ALB (g/dl	0.163	0.806	0.595 – 1.091	0.227	0.829	0.612 – 1.123
ALP ()	0.878	0.971	0.662 – 1.422	0.943	1.014	0.697 – 1.474
AAPR (≥0.64, <0.64)	0.001	0.510	0.338 – 0.770	0.003	0.536	0.364 – 0.818
TNM stage (I, II, III)	<0.001	1.756	1.494 – 2.065	<0.001	1.747	1.486 – 2.055

AAPR, albumin‐to‐alkaline phosphatase ratio; ALB, albumin; ALP, alkaline phosphatase; CI, confidence interval; DFS, disease‐free survival; Hb, hemoglobin; HR, hazard ratio; LDH, lactate dehydrogenase; OS, overall survival; WBC, white blood cell.

### Comparison of AAPR‐TNM and TNM staging systems

**Figure 5:**
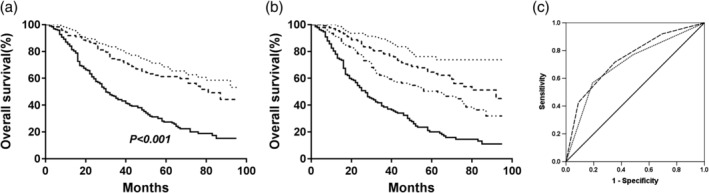
Kaplan‐Meier curves of OS. (**a**) Effect of the TNM staging system on the OS in all NSCLC patients (AUC = 0.706, 95% CI = 0.660–0.752); TNM (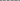) I, (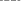) II, and (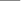) III. (**b**) Effect of the AAPR‐TNM system on OS in all NSCLC patients (AUC = 0.742, 95% CI = 0.699–0.785). AAPR‐TNM (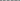) 1, (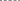) 2, (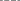) 3, and (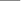) 4. (**c**) ROC analysis of the TNM staging system and the AAPR‐TNM system in OS prediction. (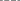) AAPR‐TNM, (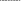) TNM, and (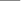) Reference line.

To obtain more reliable outcomes, we integrated the AAPR evaluation into the TNM staging system to establish the AAPR‐TNM system. In the traditional TNM staging system, the 5‐year OS rates of patients with stage I, II, or III disease were 67.5%, 59.8% and 27.4%, respectively (*P* < 0.001, Fig 5a). In the novel AAPR‐TNM staging system, the 5‐year OS rates of patients with grade 1, 2, 3, or 4 disease were 73.8%, 63.4%, 49.3%, and 20.1%, respectively (*P* < 0.001, Fig 5b). According to our results, the novel AAPR‐TNM system could classify the patients into four independent groups. The ROC curves further revealed that the AUCs of the AAPR‐TNM system and TNM staging system were 0.742 (95% CI = 0.699–0.785) and 0.706 (95% CI = 0.660–0.752), respectively. Significance was clearly identified (z = 3.316, *P* = 0.001, Fig 5c). Additionally, for OS, the AAPR‐TNM system presented a significantly larger χ^2^ value than the TNM staging system according to the LRT results (112.4 vs. 89.2, respectively, *P* < 0.01). Moreover, the AAPR‐TNM system yielded a dramatically smaller Akaike information criterion (AIC) value than the TNM staging system (2955 vs. 2977, respectively, *P* < 0.01), suggesting that in OS prediction, the AAPR‐TNM system was superior to the TNM staging system.

## Discussion

For decades, due to the convenient and economical features of serum biomarkers, many investigators have set out to identify potential prognostic markers in the routine biochemical and blood tests of cancer patients. Multiple biomarkers in serum, such as the Glasgow Prognostic Score (GPS) and neutrophil to lymphocyte ratio (NLR), have been widely revealed as effective prognostic prediction factors in cancer progression.^13^, ^14^, ^15^, ^16^ In the present study, we explored the potential prognostic value of AAPR, a novel prognostic parameter, in 496 NSCLC patients. We initially assessed the value of the preoperative AAPR as a promising and effective prognostic factor for NSCLC patients after curative surgery. We also revealed that the AAPR‐TNM system was superior to the current TNM system in OS prediction.

Albumin, the most abundant protein in serum, is specifically synthesized in liver tissues. Recently, ALB has been used to assess the nutritional status of the body and was found to function in maintaining DNA replication and promoting cell proliferation.^17^ A previous study showed that ALB can exert antioxidant effects against carcinogens and regulate systemic and organ‐ or tissue‐specific immune responses.^18^ Therefore, a low ALB level, which is a biomarker for malnutrition, indicates decreased human defense mechanisms, leading to a poor response to anticancer therapies.^19^ The clinical significance of ALB in multiple cancers, including NSCLC, has been widely explored.^6^ Recently, Miura *et al*. assessed 556 NSCLC patients and confirmed that the preoperative serum ALB level is a critical prognostic factor for OS and recurrence‐free survival (RFS).^6^

As a phosphate monoester hydrolase, ALP plays a role in catalyzing hydrolysis and removing phosphate groups under alkaline conditions.^20^ ALP is abundant in multiple tissues throughout the body; however, it is present at particularly high levels in the placenta, bone, liver, and bile duct.^21^ The serum ALP level increases in some pathological conditions, especially bone metastases. Therefore, ALP has been extensively used to screen patients for bone metastasis. Some studies have shown that the baseline ALP level and changes in the ALP level can predict treatment effects and survival in bony metastatic cancers.^22^, ^23^, ^24^ After analyzing 168 lung cancer patients, Zhang *et al*. reported that the serum ALP level is a prognostic factor for bone metastases in lung cancer and that a higher serum ALP level is associated with shorter survival.^24^

Hence, both ALB and ALP might play major roles in tumor progression. Derived from ALB and ALP, the AAPR might expand the prognostic value of these molecules, especially by reflecting the unfavorable effects of a low ALB level and a high ALP level. First reported by Anthony *et al*. the AAPR was found to be an independent prognostic factor for OS and DFS in patients with HCC regardless of the treatment approach.^7^ Since then, the AAPR has been proven to be a significant prognostic predictor for nasopharyngeal carcinoma, SCLC, and upper tract urothelial carcinoma (UTUC), whereas it has not yet been explored in patients with NSCLC.^8^, ^9^, ^10^

Our study is the first to evaluate the prognostic value of the preoperative AAPR in NSCLC patients who underwent surgical treatment. Seven variables were entered in the multivariate analyses, and interestingly, we found that decreased AAPR was independently correlated with poor prognosis in NSCLC patients. As far as we know, no clear studies have explored the roles of AAPR in different subgroups in various cancers. Our results also demonstrated that in lung cancer patients, the preoperative AAPR could predict the prognosis and classify these patients into two independent groups before surgery. When we compared the effects of the AAPR on patients with TNM stage I, II or III disease separately, significant correlations between the preoperative AAPR and both DFS and OS were found. Additionally, we combined the AAPR with the TNM staging system and found that the AAPR‐TNM system was superior to the TNM staging system for OS prediction. The AAPR‐TNM system could separate all NSCLC patients into four independent groups, which might be useful for clinical decision‐making. However, additional studies are needed in the future to confirm the practical utility of the AAPR‐TNM system.

However, the present study did have some limitations. First, this was a retrospective study, and all patients were from a single centre, which may have caused selection bias. Second, although we tried to reduce confounding influences, serum ALB and ALP levels can be affected by unknown factors. Third, the threshold value of the AAPR was identified by ROC curve analysis in our study. However, different studies have revealed specific AAPR cut‐off values. Whether these cut‐off values can be used in other independent cohorts requires further study. As a novel index, a low AAPR may indicate inactive immune reactivity and poor nutrition, which may lead to poor survival. However, basic studies are needed to clarify the underlying mechanisms of the correlation between the AAPR and prognosis.

In summary, we demonstrated that the preoperative AAPR is a potentially useful and reliable factor for predicting DFS and OS in patients with NSCLC. The incorporation of the AAPR into the current TNM staging system could divide NSCLC patients into four independent groups and help clinicians accurately predict the prognosis of NSCLC patients. Nevertheless, further studies should be performed to overcome the limitations of our study and confirm our results.
